# Inkjet Printing of High-Color-Purity Blue Organic Light-Emitting Diodes with Host-Free Inks

**DOI:** 10.3390/molecules29092147

**Published:** 2024-05-05

**Authors:** Hui Fang, Jiale Li, Shaolong Gong, Jinliang Lin, Guohua Xie

**Affiliations:** 1 Sauvage Center for Molecular Sciences, Hubei Key Laboratory on Organic and Polymeric Optoelectronic Materials, Department of Chemistry, Wuhan University, Wuhan 430072, China; 2021202030158@whu.edu.cn (H.F.); jialeli@whu.edu.cn (J.L.); 2020282030166@whu.edu.cn (J.L.); 2 The Institute of Flexible Electronics (Future Technologies), Xiamen University, Xiamen 361005, China

**Keywords:** inkjet printing, thermally activated delayed fluorescence, organic light-emitting diodes, host-free inks

## Abstract

Inkjet printing technology offers a unique approach to producing direct-patterned pixels without fine metal masks for active matrix displays. Organic light-emitting diodes (OLEDs) consisting of thermally activated delayed fluorescence (TADF) emitters facilitate efficient light emission without heavy metals, such as platinum and iridium. Multi-resonance TADF molecules, characterized by their small full width at half maxima (FWHM), are highly suitable for the requirements of wide color-gamut displays. Herein, host-free TADF inks with a low concentration of 1 mg/mL were developed and inkjet-printed onto a seeding layer, concurrently serving as the hole-transporting layer. Attributed to the proof-of-concept of host-free inks printed on a mixed seeding layer, a maximum external quantum efficiency of 13.1% (improved by a factor of 21.8) was achieved in the inkjet-printed OLED, with a remarkably narrow FWHM of only 32 nm. Highly efficient energy transfer was facilitated by the effective dispersion of the sensitizer around the terminal emitters.

## 1. Introduction

Organic light-emitting diodes (OLEDs) are recognized as the most promising energy-saving and environmentally friendly solid-state lighting and full-color display technology due to the advantages related to their high efficiency, wide viewing angle, high contrast, ultra-small thickness, flexibility, and transparency [1,2,3]. Most OLED panels are mass-produced via ultra-high vacuum (<10^−6^ mbar) thermal evaporation processes and have multilayer architecture composed of many functional layers. These high-vacuum environments and fine masks require a high fabrication cost for mass production. Solution-processing techniques are more competitive in large-area, flexible and low-cost OLED products. Inkjet printing (IJP) is a fast, accurate, mask-free deposition process that enables high material utilization and direct patterning ability, rendering inkjet printing one of the more cost-effective deposition methods. It is clear that employing IJP to create OLED panels is preferable, without using expensive fine metal masks and vacuum thermal evaporation [4].

Benefiting from the rapid development of the display industry, significant progress has been made in relation to OLEDs based on thermally activated delayed fluorescence (TADF) materials in both the academic and industrial fields. Unlike heavy-metal phosphorescent materials, purely organic TADF materials involve a lower production cost and provide a more efficient way to utilize all of the electrically generated excitons. Additionally, purely organic TADF emitters, including the twisted donor–acceptor (D-A)-type emitters and multi-resonance TADF (MR-TADF) emitters [3], typically feature small enough singlet–triplet energy gaps (ΔE_ST_s) to harvest electrically generated singlet and triplet excitons by means of reverse intersystem crossing (RISC) and thus realize 100% internal quantum efficiency (IQE) [5,6].

However, donor–acceptor (D-A) TADF compounds typically show very broad emission due to their charge-transfer nature, since the D and A units are connected by single bonds, resulting in the broad distribution of accessible geometries in the excited state [2,7]. This results in poor color purity. The current state-of-the-art MR-TADF molecules are usually grafted/substituted with the push–pull electron groups on the fixed MR parent core to tune the emission. For instance, boron (B)/nitrogen (N) atoms are distributed on the opposite sites to induce the separated distributions of the highest occupied molecular orbitals (HOMOs) and the lowest unoccupied molecular orbitals (LUMOs) [8,9]. Due to the reduced excited-state relaxation, MR-TADF emitters can achieve high efficiencies and small FWHMs simultaneously [10,11]. Therefore, their emissions with the FWHM (<40 nm) could be similar to those of quantum-dots and inorganic LEDs. Otherwise, the auxiliary design of RGB color filters is necessary [12,13]. This design strategy was initially proposed by Hatakeyama et al., sparking a research trend in the scientific community towards multi-resonance molecules. Duan et al reported highly efficient yellow-light multi-resonance molecules, achieving a narrow bandwidth of only 23 nm and a maximum external quantum efficiency (EQE) of up to 37.4% in vacuum-deposited devices [8]. Wang et al successfully synthesized a multi-resonance system incorporating C=O/N to achieve green emission, with an EQE as high as 37.2% in the vacuum-deposited devices and a narrow bandwidth of only 24 nm [14]. Although multi-resonance materials have shown remarkable performance in vacuum-deposited devices, their application in inkjet printing remains limited. The difference in the preparation methods of the emissive layer between vacuum-deposited and inkjet-printed devices lies in the fact that, through vapor deposition, extremely low concentrations of multi-resonant emissive molecules can be doped into the host material. The low-concentration doping of guest molecules into the host material primarily serves to suppress the aggregation of emissive molecules, reducing the severe non-radiative transitions caused by π–π interactions between guest molecules, which lead to luminescence quenching and a significant decrease in the photoluminescence quantum yield (PLQY) of the emissive molecules. Typically, MR-TADF has serious aggregation-caused quenching (ACQ) effects due to the rigid structures [15].

It is very challenging to design and fabricate high-performance OLEDs with small molecules via inkjet printing. Firstly, the commercial small molecules tend to show poor solubility and terrible film morphologies via solution processing. For example, the large planar structure of MR-TADF molecule exhibits poor solubility. Additionally, phase separation, recrystallization, and coffee ring effects of the inkjet-printed small molecules worsen the device performance. Therefore, many researchers adopt a co-solvent strategy to develop the inks in order to suppress the poor film uniformity caused by the coffee ring effect [16]. Nevertheless, the uniformity of the inkjet-printed films is generally worse than that of the spin-coated films [17]. One of the main challenge of IJP OLEDs is to develop a suitable ink formulation and achieve stable ink-jetting to ensure uniform film deposition [18].

Therefore, research on achieving the efficient narrow-emission inkjet printing of TADF molecules based on single-solute IJP devices has been relatively less well explored. This is primarily due to the fact that single-solute printing inks only contain emissive guest materials, which are directly printed onto the hole injection layer. Such devices are only applicable for non-doped devices. Additionally, for emissive molecules prone to aggregation-caused quenching, single-solute inks can also lead to severe aggregation of the guest molecules. Consequently, the selection of guest molecules is limited by single-solute IJP. In order to meet the requirements of doped devices, some researchers simultaneously introduce host and guest materials into the ink. While this ensures uniform dispersion of materials in the ink, during the IJP process, occasional unexpected phase separation phenomena may occur alongside the solvent evaporation of the ink droplets. There are four key factors that affect the droplet morphology: ink density, surface tension, viscosity, and nozzle diameter [19]. Additionally, the boiling point of the ink also plays a crucial role in ensuring successful printing [20]. When using lower-boiling-point solvents as ink, the rapid evaporation of ink during the printing process can easily lead to nozzle clogging [21]. Ink formulations with the lower- and higher-boiling-solvent mixture can help to avoid rapid evaporation. Huang et al. systematically elucidated the optimization process of ink and substrate for inkjet printing, and ultimately achieved the inkjet printing of small-molecule blue OLEDs with a maximum current efficiency of 6.1 cd/A using environmentally friendly halogen-free inks [4]. The study by Katiyar et al. involved the estimation of Hansen solubility parameters to formulate inkjet-printed ink with an iridium complex-based phosphorescent material. The fabricated devices exhibit a maximum current efficiency of 6.4 cd/A and a maximum luminance of 5781 cd/m^2^.

Additionally, large-area OLEDs with a size of 80 × 80 mm^2^ were simultaneously fabricated by using IJP technology for the emissive layer [22]. Cui et al. successfully designed and synthesized three hosts to optimize the performance of the IJP device with a maximum external quantum efficiency (EQE) of 11.0%, which was one of the highest among the reported TADF-OLEDs fabricated via the IJP approach [23].

In this contribution, we developed a host-free ink strategy, where the host materials were spin-coated as a seeding layer prior to the inkjet-printed emitters. We screened a conventional fluorescent polymer poly(9-vinylcarbazole) (PVK) and the host 1,3-bis(carbazol-9-yl) benzene (mCP) to construct a binary-blended hole-transporting layer (HTL). The MR-TADF emitter with bis(di(t-butyl)carbazolyl)phenylene as a parent skeleton, i.e., BCzBN, featuring a large and rigid π-conjugated core skeleton was used as the guest emitter, which possessed a high PLQY, a small FWHM, and improved solubility. The IJP devices were prepared with the binary ink of SBA-2DPS:BCzBN, where SBA-2DPS was used to sensitize BCZBN. This resulted in a maximum EQE of 10.1%, which was over 4-fold higher than that (2.5%) of the device with the single-component solute of BCzBN. To further enhance device efficiency and reduce the aggregation quenching of BCzBN, we optimized the ink composition, and a maximum EQE of 13.1% was achieved, accompanied by a small FWHM of only 32 nm. To the best of our knowledge, this represents the highest EQE for inkjet-printed devices based on MR-TADF molecules.

## 2. Results and Discussion

**Figure 1:**
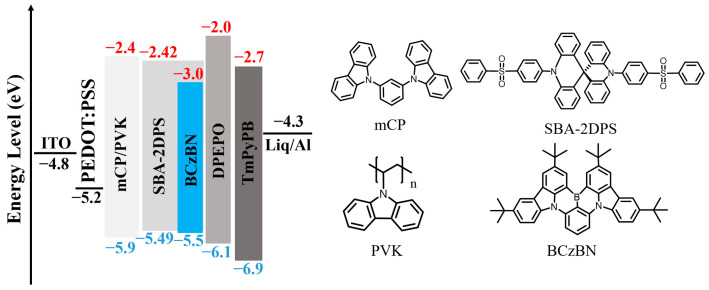
Schematic diagram of energy level alignment of the device and chemical structures of the key materials used in the IJP devices. The sky-blue columns represent the luminescent guest molecule. The values of the energy levels were adopted from the literature.

To prevent direct contact between the emissive layer and the hole-injection layer poly(3,4-ethylenedioxythiophene):poly(styrenesulfonic acid) (PEDOT:PSS), we employed the mixed HTL with the small-molecule fluorescent material mCP and PVK, which could avoid being completely dissolved by the ink used to print the host-free emitters. Moreover, PVK is key to addressing the issue of poor film uniformity of mCP and the problem of post-annealing recrystallization due to a low glass transition temperature (T_g_ < 60 °C) of mCP. To balance charge transport, we employed mCP:PVK at a ratio of 80:20 (wt./wt.) in the HTL. Figure 1 illustrates the molecular structures of the HTL, the sensitizer SBA-2DPS, and the terminal emitter BCzBN, along with the energy level diagram of the device.

**Figure 2:**
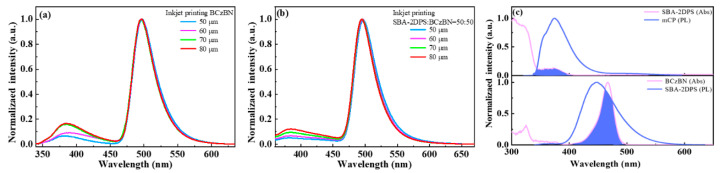
Normalized PL spectra of (**a**) BCzBN and (**b**) SBA-2DPS:BCzBN films printed on the HTL with different dot spacings of 50, 60, 70 and 80 μm. (**c**) Normalized PL spectra of mCP and SBA-2DPS films and UV-vis absorption of the SBA-2DPS and BCzBN solution (10^−5^ M).

As shown in Figure 2a,b, the steady-state PL spectra of the IJP BCzBN and SBA-2DPS:BCzBN = 50:50 (wt./wt.) onto the HTL exhibit the characteristic emission of BCzBN with the residual emission from the HTL. Due to the good overlap of the absorption of the terminal emitter BCzBN and the emission of the sensitizer SBA-2DPS (see Figure 2c), no residual emission from the sensitizer is detected (see Figure 2b) due to the complete energy transfer. At the same time, it can also be observed that energy transfer from the hole transport layer mCP to the sensitizer is achievable. The emission band ranging from 350 to 450 nm originating from PVK is mainly attributed to the emission from the gaps between the adjacent droplets.

The steady PL intensity of the IJP BCzBN on the HTL is shown in Figure S1. At a dot spacing of 80 μm, the highest intensity is achieved. As the spacing decreases, the droplets merge with each other, causing significant solute aggregation and fluorescence quenching. This leads to a decrease in spectral intensity. Figure S2 shows the steady PL intensity of SBA-2DPS:BCzBN films under identical testing condition. The intensity of the IJP film is significantly higher when a sensitizer is introduced into the ink compared with the single-solute ink, indicating efficient energy transfer and reduced aggregation quenching of the guest emitter BCzBN by increasing the intermolecular distance using the sensitizer. As shown in Figures S3–S5, the droplets printed with a dot spacing of 80 μm exhibit individual and separated round patterns. In contrast, the spin-coated film is quite flat and smooth (Figure S6b).

**Figure 3:**
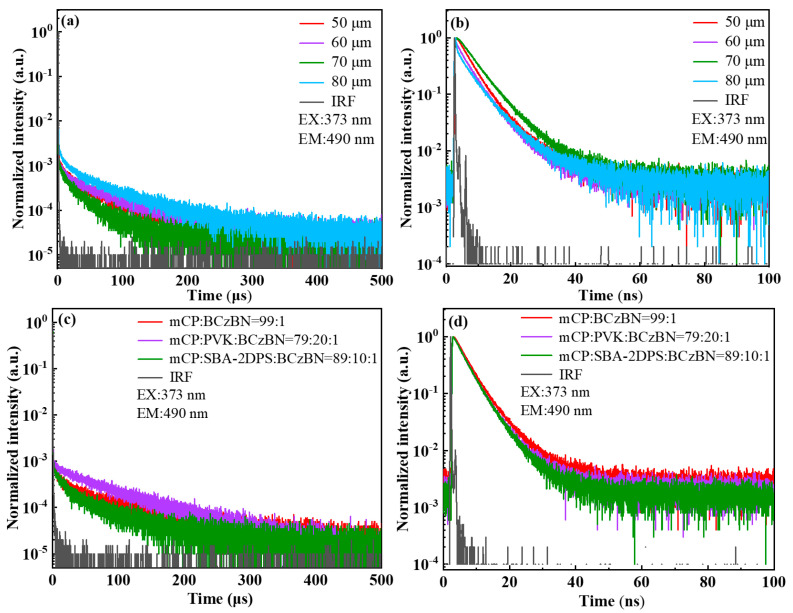
Transient PL spectra of the IJP films of SBA-2DPS:BCzBN films fabricated with dot spacings of 50, 60, 70, and 80 μm detected at long (**a**) and short (**b**) time scales, as well as spin-coated films detected at long (**c**) and short (**d**) time scales.

To investigate the photophysics of the films, the time-resolved PL spectra of the printed and spin-coated films were compared (see Figure 3 and Table S1). The delayed lifetimes of the printed films from the dual solute are around 60 μs, nearly independent of the dot spacing, since the emission mainly originated from BCzBN. However, the delayed lifetimes of the spin-coated films with only the guest emitter BCzBN are approximately 90 μs. It is surprising that the spin-coated film with the SBA-2DPS:BCzBN mixture (Figure 3c) displayed a delayed lifetime of 57.5 μs. Therefore, the reduction in the delayed lifetime stems from the fast and efficient energy transfer process from the sensitizer SBA-2DPS to the guest molecule BCzBN.

**Figure 4:**
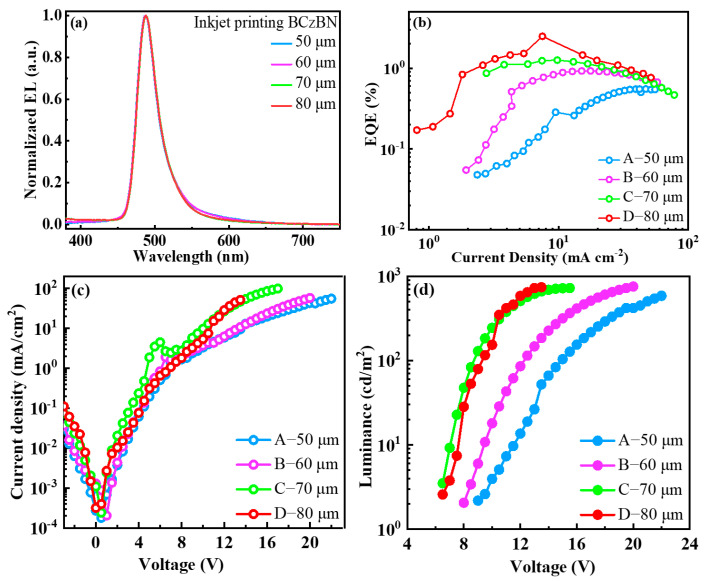
(**a**) Normalized EL spectra. (**b**) External quantum efficiency versus current density curves of the devices with different printed dot spacings. (**c**) Current density–voltage curves. (**d**) Luminance–voltage curves.

To elucidate the electroluminescent performance of the inkjet-printed OLEDs, four different devices with the dot spacings of 50 (Device A), 60 (Device B), 70 (Device C), and 80 μm (Device D) were respectively fabricated with the structures of indium tin oxide (ITO)/PEDOT:PSS (40 nm)/mCP:PVK (80:20, 40 nm)/IJP film/bis[2-(diphenylphosphino)phenyl]ether oxide (DPEPO) (10 nm)/1,3,5-tri(m-pyrid-3-yl-phenyl)benzene (TmPyPB) (50 nm)/lithium 8-hydroxyquinolinolate (Liq) (1 nm)/Al (100 nm). Here, the IJP films consisted of host-free and single-solute BCzBN dissolved in a co-solvent of cyclohexanone and N-methylpyrrolidone at a concentration of 1 mg/mL. Figure 4 shows the current–voltage–luminance characteristics of the OLEDs. The electroluminescence spectra of the IJP devices exhibit an identical small FWHM of only 32 nm, ensuring high color purity. Unlike the PL spectra of the IJP films, there is no residual emission from the HTL.

As shown in Table 1, the maximum EQE of the device D reached 2.5%. However, the maximum EQE of the device with a printing dot spacing of 50 μm was only 0.6%. With the increase in dot spacing, the EQE of the device increases. These results demonstrate that employing a single-solute strategy is not efficient enough due to the pronounced aggregation quenching effects of the MR-TADF emitters.

**Figure 5:**
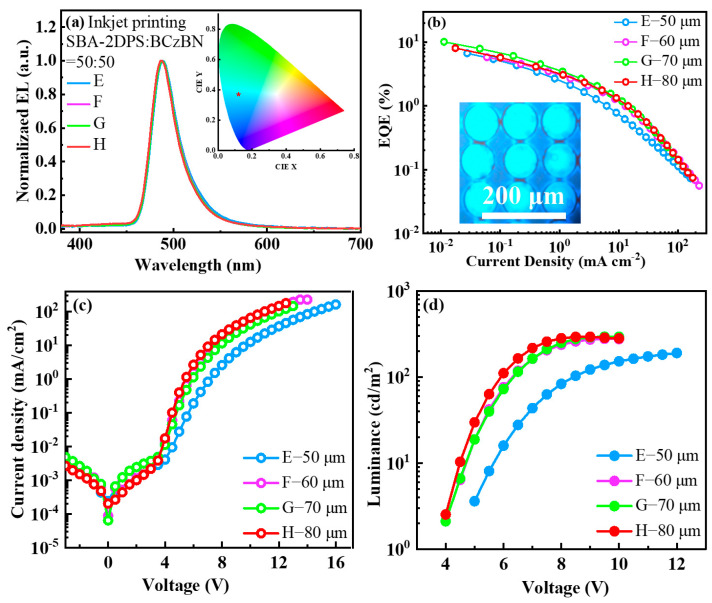
(**a**) Normalized EL spectra. Inset: The CIE 1931 color coordinates of the IJP devices. (**b**) EQE versus current density curves. Inset: Microscopic photograph of the IJP device under electroluminescence. (**c**) Current density–voltage curves. (**d**) Luminance–voltage curves.

Therefore, to serve as a dispersant and reduce the aggregation of the guest molecule BCzBN, the TADF sensitizer SBA-2DPS was introduced into the ink. An ink mixture of SBA-2DPS:BCzBN = 50:50 (wt./wt.) was prepared to fabricate the emissive layer again with a dot spacing of 50 (Device E), 60 (Device F), 70 (Device G), and 80 μm (Device H), respectively. As shown in Figure 5a, the electroluminescent spectra of all of the devices displayed no residual emission from the sensitizer and HTL (see Figure 2), indicating an improved efficient energy transfer process under electrical excitation. At the same time, a relatively small FWHM is maintained, ensuring the high color purity of the dual-solute device. Moreover, for the printed dual-solute devices, the leakage current was significantly reduced, indicating excellent metal/organic contact (see Figure 5c). As illustrated in Figure 5b, the device G achieved a maximum EQE of 10.1%, which is four times higher than that of the single-solute device D (2.5%). The inset of Figure 5b shows a microscopic photograph of the device under electroluminescence. The droplets display bright sky-blue emissive patterns. The introduction of the sensitizer led to a significant reduction in the driving voltage. For instance, the operation voltages at a luminance of 10 cd/m^2^ were found to be 5.6, 4.6, 4.6, and 4.5 V, respectively, for the devices E, F, G, and H (see Table 2).

To further enhance the device efficiency, the proportion of the sensitizer SBA-2DPS was increased to 70 wt.%, while the optimized dot spacing was fixed at 50 μm. The emissive layer was inkjet-printed with the dual-solute ink of SBA-2DPS:BCzBN = 70:30 (wt./wt.) at a concentration of 1 mg/mL in the co-solvent system. As illustrated in Figure S8a, despite a further reduction of BCzBN, there is no noticeable change in the electroluminescent spectrum, indicating that efficient energy transfer remains in the emissive layer. Simultaneously, a small FWHM of only 32 nm was achieved. The device with a dot spacing of 50 μm reached a maximum EQE of 13.1% (see Figure S8b and Table S2), which is comparable with the spin-coated devices shown in Figure S9 and Table S3. It is worth mentioning that the overall EL performance of these IJP OLEDs with the host-free inks surpasses those of the recently reported state-of-the-art devices (see Figure S10) [22,23,24,25,26,27]. Based on our dual-solute and host-free inks strategy, cost-effective inkjet-printed OLEDs with a simplified architecture are achievable.

## 3. Experimental Section

The PL and UV absorption spectra were measured using a Hitachi F-4600 fluorescence spectrophotometer (Tokyo, Japan) and Shimadzu UV-2700 UV-VIS spectrophotometer (Tokyo, Japan), respectively. The transient PL decay curves were obtained using a FluoTime 300 (PicoQuant GmbH, Berlin, Germany) with a Picosecond Pulsed UV-LASER (LASER375, Berlin, Germany) as the excitation source.

### 3.1. OLED Fabrication and Characterization

The pre-patterned ITO glass substrates were cleaned in an acetone and ethanol ultrasonic bath continuously. Afterwards, the substrates were dried with N_2_ and loaded into a UV-ozone chamber for a 20 min treatment. A ca. 40 nm thick PEDOT:PSS was spin-coated onto the ITO substrate and then baked at 120 °C for 10 min. The hole-transporting layer was spin-coated directly on the PEDOT:PSS, followed by a baking process at 120 °C for 10 min. Except for the emitting layer, the electron-transporting layer and the electron-injecting layer, followed by the Al cathode used in the IJP devices, were evaporated at a pressure of 10^−6^ mbar in a high-vacuum chamber, consecutively. Finally, the devices were encapsulated in UV-curable epoxy in a glovebox before being measured in ambient air. The electroluminescent properties of the devices were measured using a PR735 SpectraScan Spectroradiometer (PhotoResearch, Syracuse, NY, USA) combined with a Keithley 2400 source meter unit (Cleveland, OH, USA).

### 3.2. Inkjet Printing of the Emitting Layer

Other than the emitting layer, the preparation process of the other functional layers of inkjet-printed OLED was consistent with that of spin-coating OLED. Prior to inkjet printing, the mixed seed layer of mCP (80 wt.%):PVK (20 wt.%) was prepared by spin-coating directly on PEDOT:PSS. Then, the host-free inks comprising the TADF emitter was inkjet printed on the mCP:PVK layer with the dot spacing of 50, 60, 70, and 80 μm, respectively. The temperature of the substrate was fixed at 40 °C. In this experiment, single-nozzle printing was employed to achieve simultaneous coverage of the active areas, printing a rectangular pattern measuring 9 mm × 7.5 mm. The peak voltage of the printing waveform was set to 18 V, with a frequency of 960 Hz, and the printing waveform is shown in Figure S11. The inkjet printing operation was carried out with an MP1100 electronics printer from Shanghai MiFang Electronic Technology (Shanghai, China). After setting all parameters, printing commenced. Subsequently, the printed samples were transferred to a glovebox for annealing at 50 °C for 10 min to remove any residual solvents. The subsequent evaporation and encapsulation processes were identical to those used in the spin-coating device fabrication process.

## 4. Conclusions

In summary, the common small-molecule host mCP and the fluorescent polymer PVK were introduced as the seeding layer of the inkjet-printed OLEDs, physically isolating the emitters prepared from the host-free inks. This separation mitigates the concentration quenching of the multi-resonant TADF emitters. The adoption of the highly efficient TADF molecule SBA-2DPS as a sensitizer is crucial to enhancing the electroluminescence, since it is powerful in reducing exciton quenching and facilitating highly efficient energy transfer. Ultimately, the inkjet-printed devices achieved a considerably high external quantum efficiency of 13.1%, representing an improvement factor of ca. 22, compared with the inkjet-printed devices consisting of the single-solute BCzBN fabricated with the same parameter (dot spacing of 50 μm). This strategy would be universal for the inkjet-printed devices with any other host-free emitters/inks.

## Figures and Tables

**Table 1:** Comparison of the EL performances of the inkjet printing devices with different printing spacings.

Device	λ_max_ ^a^ (nm)	FWHM ^b^ (nm)	V_on_ ^c^ (V)	CE_max_ ^d^ (cd/A)	EQE_max_ ^e^ (%)	CIE(x,y) ^f^
A	488	32	11.5	1.1	0.6	(0.14, 0.38)
B	488	32	9.5	2.1	0.9	(0.14, 0.38)
C	488	32	7.3	2.5	1.3	(0.14, 0.39)
D	488	32	7.5	4.6	2.5	(0.12, 0.38)

^a^ Peak wavelength of the electroluminescent spectrum. ^b^ Full width at half maximum of the electroluminescent spectrum. ^c^ Driving voltage at a brightness of 10 cd/m^2^. ^d^ Maximum current efficiency. ^e^ Maximum external quantum efficiency. ^f^ Commission Internationale de I’Eclairage 1931 color coordinates.

**Table 2:** Comparison of the EL performances of the inkjet printing devices with different printing spacings.

Device	λ_max_ ^a^ (nm)	FWHM ^b^ (nm)	V_on_ ^c^ (V)	CE_max_ ^d^ (cd/A)	EQE_max_ ^e^ (%)	CIE(x,y) ^f^
E	488	32	5.6	8.7	4.5	(0.13, 0.39)
F	488	32	4.6	10.7	5.8	(0.13, 0.37)
G	488	32	4.6	18.7	10.1	(0.12, 0.37)
H	488	32	4.5	14.4	8.1	(0.12, 0.35)

^a^ Peak wavelength of the electroluminescent spectrum. ^b^ Full width at half maximum of the electroluminescent spectrum. ^c^ Driving voltage at the brightness of 10 cd/m^2^. ^d^ Maximum current efficiency. ^e^ Maximum external quantum efficiency. ^f^ Commission Internationale de I’Eclairage 1931 color coordinates.

## Data Availability

The data presented in this investigation is available from the corresponding authors.

## Supplementary Materials

The following supporting information can be downloaded at: https://www.mdpi.com/article/10.3390/molecules29092147/s1, Figure S1: PL spectra of BCzBN films printed on the hole transporting layer with the different dot-spacings of 50, 60, 70, and 80 μm, respectively. The spectrum of the hole-transporting layer was provided as reference; Figure S2: PL spectra of SBA-2DPS:BCzBN = 50:50 (wt./wt.) co-doped films on the hole transporting layer with the different dot spacings of 50, 60, 70, and 80 μm, respectively; Figure S3: Micrographs of the inkjet-printed BCzBN films with different dot spacing: (a) 50 μm, (b) 60 μm, (c) 70 μm, and (d) 80 μm; Figure S4: Surface profiles the inkjet-printed films with different dot spacings: (a) 50 μm, (b) 60 μm, (c) 70 μm, and (d) 80 μm; Figure S5: Micrographs of the inkjet-printed SBA-2DPS:BCzBN=50:50 films with different dot spacing: (a) 50 μm, (b) 60 μm, (c) 70 μm, and (d) 80 μm; Figure S6: Surface profiles of the inkjet-printed film (a) and the spin-coated film (b); Figure S7: (a) Thickness profile of PEDOT:PSS and seeding layer measured from a Bruker Dektak Profiler. (b) Surface profile of inkjet-printed SBA-2DPS:BCzBN = 50:50 film with a dot spacing of 80 μm; Figure S8: (a) Normalized EL spectra of the inkjet-printed devices with dual-solute ink of SBA-2DPS:BCzBN = 70:30 (wt./wt.). (b) EQE versus current density curves for devices at different printing spacings, (c) Current density–voltage curves. (d) Luminance–voltage curves; Figure S9: (a) Normalized EL spectra of the spin-coated devices. (b) External quantum efficiency versus current density curves for the spin-coated devices. (c) Current density–voltage curves. (d) Luminance–voltage curves; Figure S10: Figure of merit of EQE and FWHM of OLEDs based on inkjet printing reported in literature; Figure S11: Inkjet printing waveform; Table S1: Prompt and delayed fluorescence lifetime constants of the printed and spin-coated films; Table S2: Comparison of the EL Performances of the inkjet-printed devices with the dual-solute ink of SBA-2DPS:BCzBN = 70:30 (wt./wt.); Table S3: Comparison of the EL Performances of the spin-coated devices with different EML: mCP:BCzBN = 99:1 for Devcie M, mCP:PVK:BCzBN = 79:20:1 for Devcie N, mCP:SBA-2DPS:BCzBN = 94:5:1 for Devcie O, and mCP:SBA-2DPS:BCzBN = 89:10:1 for Devcie P.
