# Supplementary material for: Inkjet Printing of High-Color-Purity Blue Organic Light-Emitting Diodes with Host-Free Inks
Source: Molecules. 2024 May 5;29(9):2147. doi: 10.3390/molecules29092147 (PMC11085598; doi:10.3390/molecules29092147)
Supplement: Supplementary file 1 [file molecules-29-02147-s001.zip › molecules-2950499-supplementary.pdf]

molecules

Inkjet printing of high-color-purity blue organic light-emitting diodes with host-free inks

Hui Fang [1], Jiale Li [1], Shaolong Gong [1]*, Jinliang Lin [1,2] and Guohua Xie [1,2,]*

[1] Sauvage Center for Molecular Sciences, Hubei Key Laboratory on Organic and Polymeric Optoelectronic Materials, Department of Chemistry, Wuhan University, Wuhan 430072, China; 2021202030158@whu.edu.cn (H.F.); jialeli@whu.edu.cn (J.L.); 2020282030166@whu.edu.cn (J.L.)
[2] The Institute of Flexible Electronics (Future Technologies), Xiamen University, Xiamen 361005, China
* Correspondence: slgong@whu.edu.cn (S.G.) and ifeghxie@xmu.edu.cn (G.X.)

General information

The TADF materials, SBA-2DPS and BCzBN were synthesized by our group. The modified PEDOT:PSS was purchased from Jinghang Guangdian (Shenzhen) Corporation. The hole transporting material PVK, the host material mCP, and the electron injecting material Liq were used as purchased from Xi'an Polymer Ltd. The hole blocking material DPEPO and the electron transport material TmPyPB were purchased from Xinrunsheng (Shanghai) Corporation.

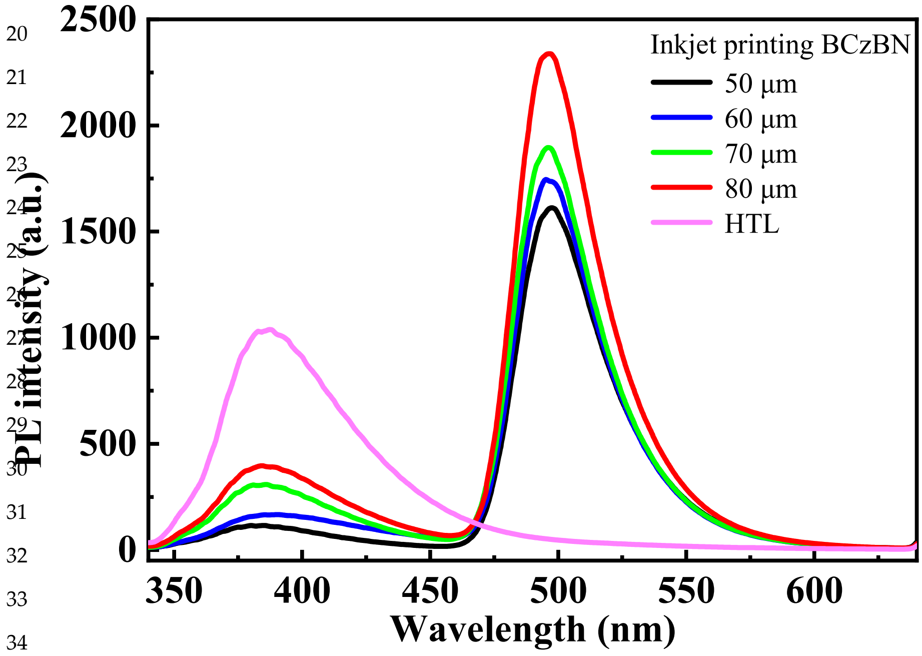

Figure S1. PL spectra of BCzBN films printed on the hole transporting layer with the different dot spacings of 50, 60, 70, and 80 μm, respectively. The spectrum of the hole transporting layer was provided as reference.

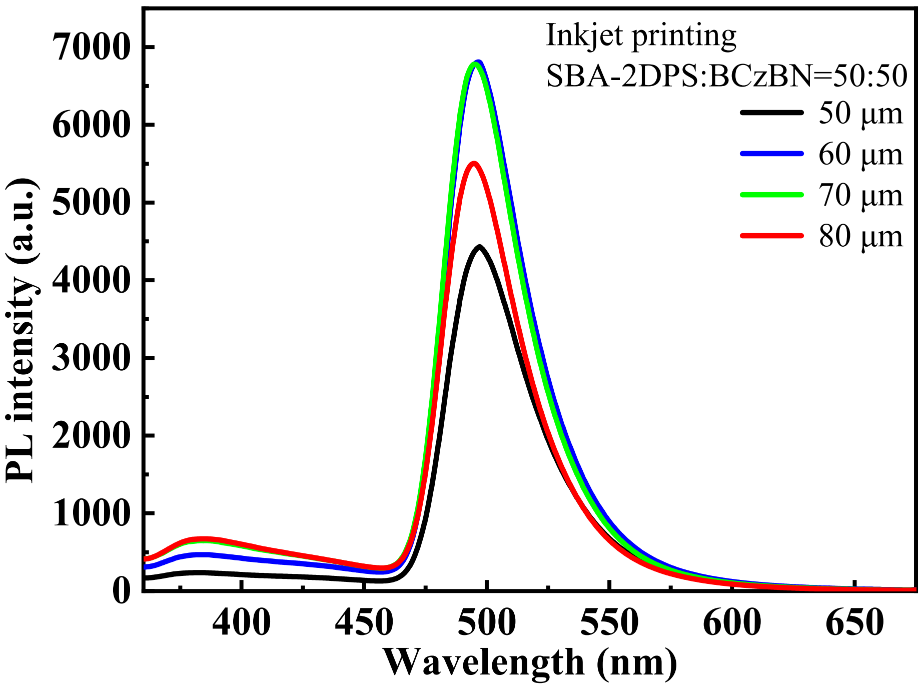

Figure S2. PL spectra of SBA-2DPS:BCzBN = 50:50 (wt./wt.) co-doped films on the hole transporting layer with the different dot spacings of 50, 60, 70, and 80 μm, respectively

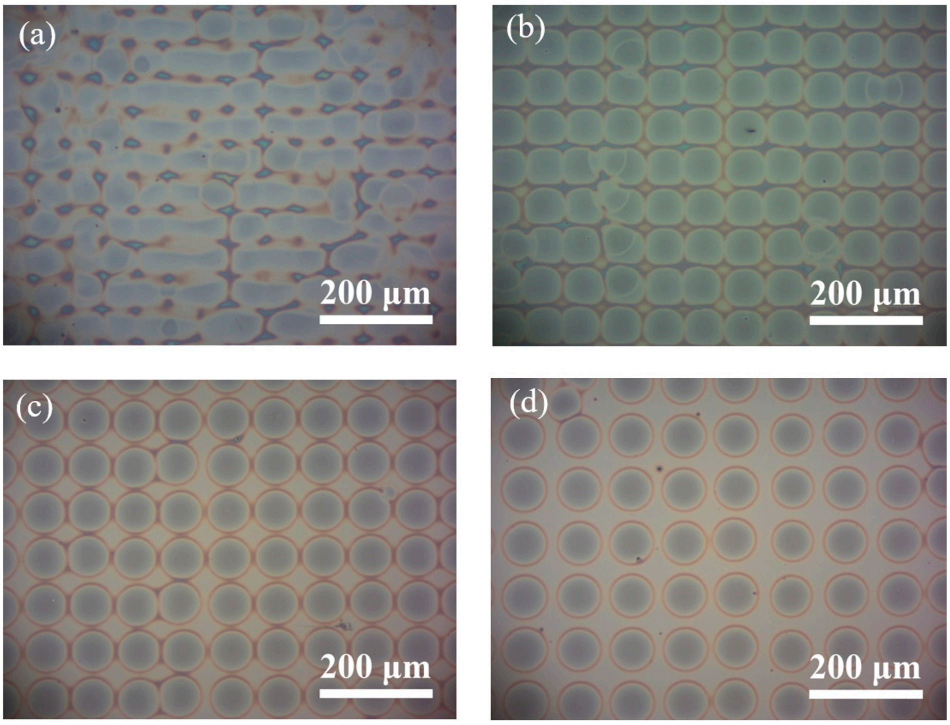

Figure S3. Micrographs of the inkjet-printed BCzBN films with different dot spacing: (a) 50 μm, (b) 60 μm, (c) 70 μm, and (d) 80 μm.

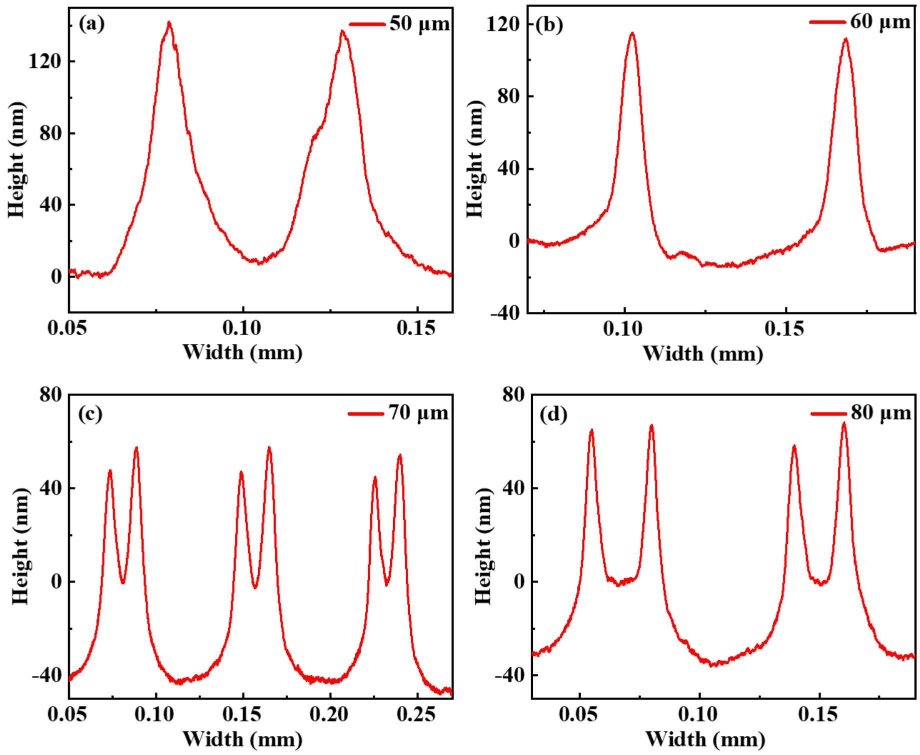

Figure S4. Surface profiles the inkjet-printed films with different dot spacings: (a) 50 μm, (b) 60 μm, (c) 70 μm, and (d) 80 μm.

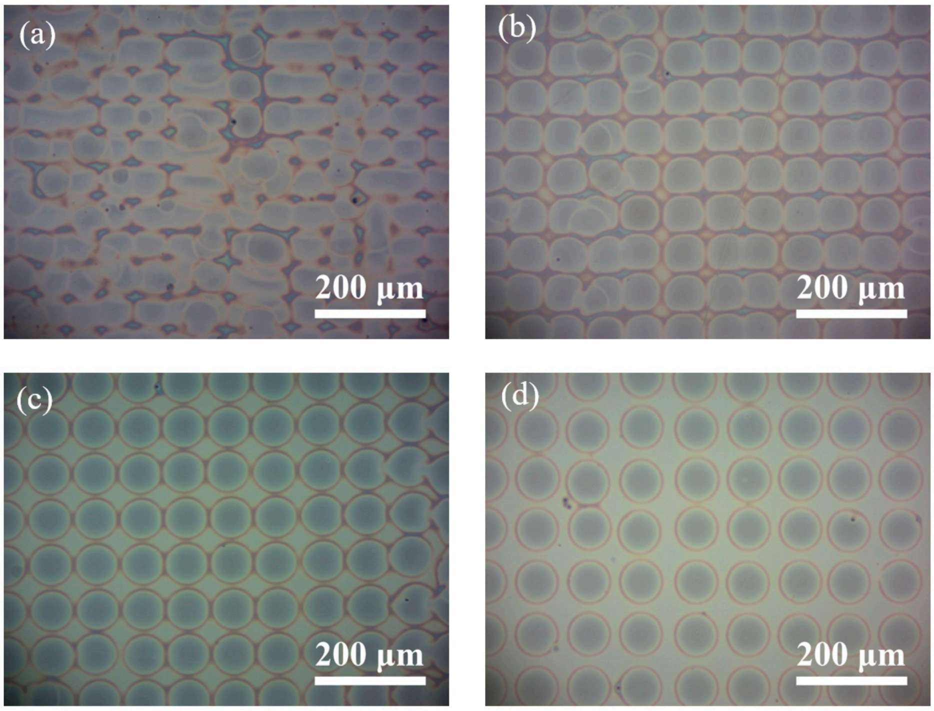

Figure S5. Micrographs of the inkjet-printed SBA-2DPS:BCzBN = 50:50 films with different dot spacing: (a) 50 μm, (b) 60 μm, (c) 70 μm, and (d) 80 μm.

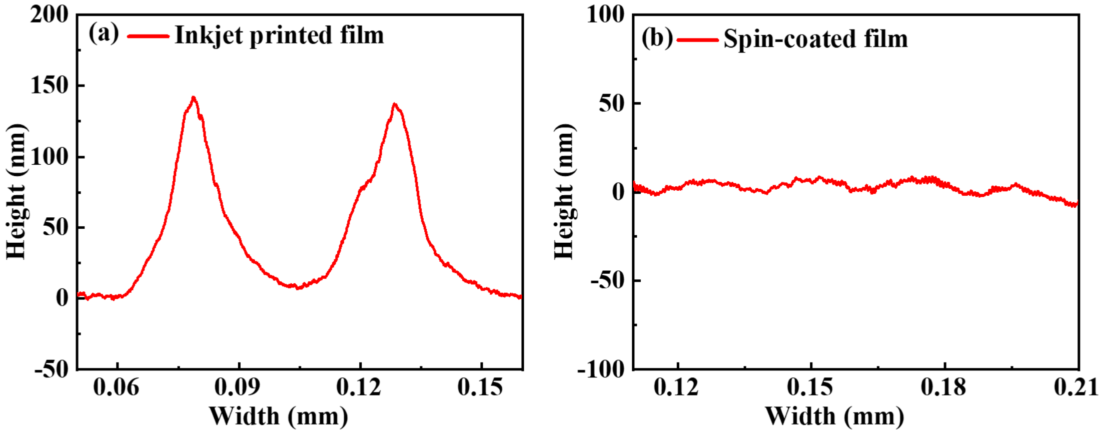

Figure S6. Surface profiles of the inkjet-printed film (a) and the spin-coated film (b).

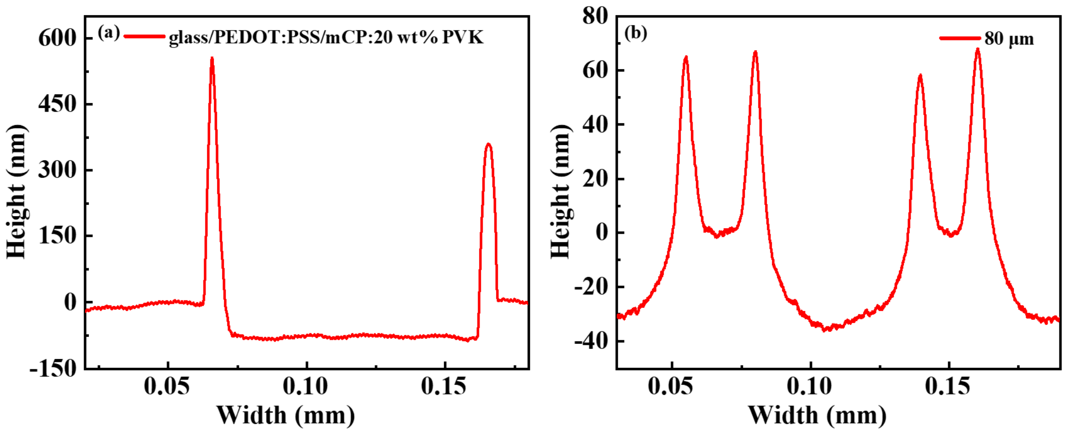

Figure S7. (a) Thickness profile of PEDOT:PSS and seeding layer measured from a Bruker Dektak Profiler. (b) Surface profile of inkjet-printed SBA-2DPS:BCzBN = 50:50 film with a dot spacing of 80 μm.

Table S1. Prompt and delayed fluorescence lifetime constants of the printed and spin-coated films.

**Inkjet-printed film SBA-2DPS:BCzBN = 70:30 (wt./wt.)**	τ_{prompt} (ns)	τ_{delay} (μs)
50 μm	5.4	58.7
60 μm	5.3	63.4
70 μm	6.6	62.6
80 μm	5.8	63.6
Spin-coated film	τ_{prompt} (ns)	τ_{delay} (μs)
mCP:BCzBN=99:1	4.9	89.0
mCP:PVK:BCzBN=79:20:1	4.5	91.4
mCP:SBA-2DPS:BCzBN=89:10:1	4.4	57.5

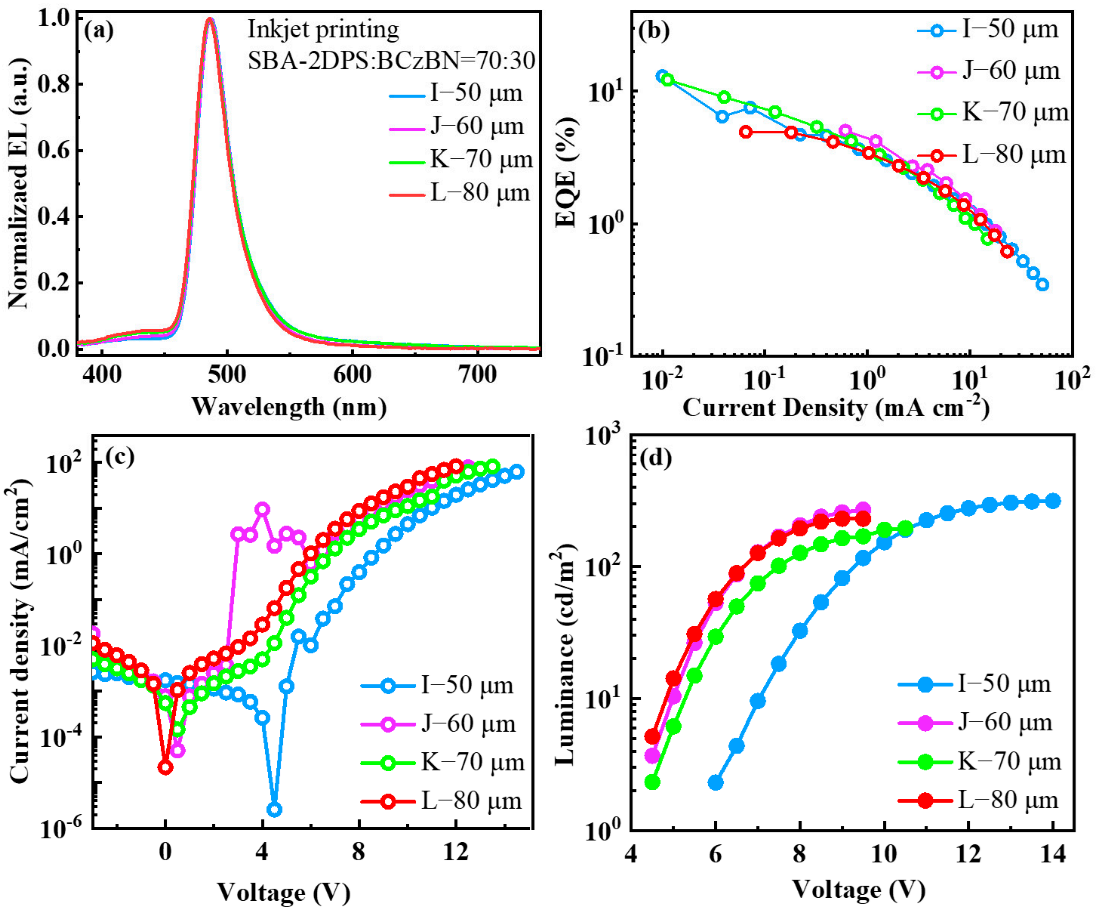

Figure S8. (a) Normalized EL spectra of the inkjet-printed devices with dual-solute ink of SBA-2DPS:BCzBN = 70:30 (wt./wt.). (b) EQE versus current density curves for devices at different printing spacings, (c) Current density−voltage curves. (d) Luminance−voltage curves.

Table S2. Comparison of the EL Performances of the inkjet-printed devices with the dual-solute ink of SBA-2DPS:BCzBN = 70:30 (wt./wt.).

device	λ_{max}[nm]	FWHM[nm]	V_{on} ^{a}[V]	CE_{max}^{b} [cd A^{-1}]	EQE_{max}^{c} [%]	CIE(x,y)^{d}
I	486	32	7.0	23.1	13.1	(0.11,0.35)
J	486	32	5.0	8.7	5.0	(0.13,0.33)
K	486	32	5.2	20.8	12.2	(0.11,0.32)
L	486	32	4.9	8.0	4.9	(0.11,0.29)

[a]Driving voltage at the brightness of 10 cd/m^2. [b]Maximum of current efficiency. [c]Maximum of external quantum efficiency. [d]The Commission Internationale de I’Eclairage 1931 color coordinates.

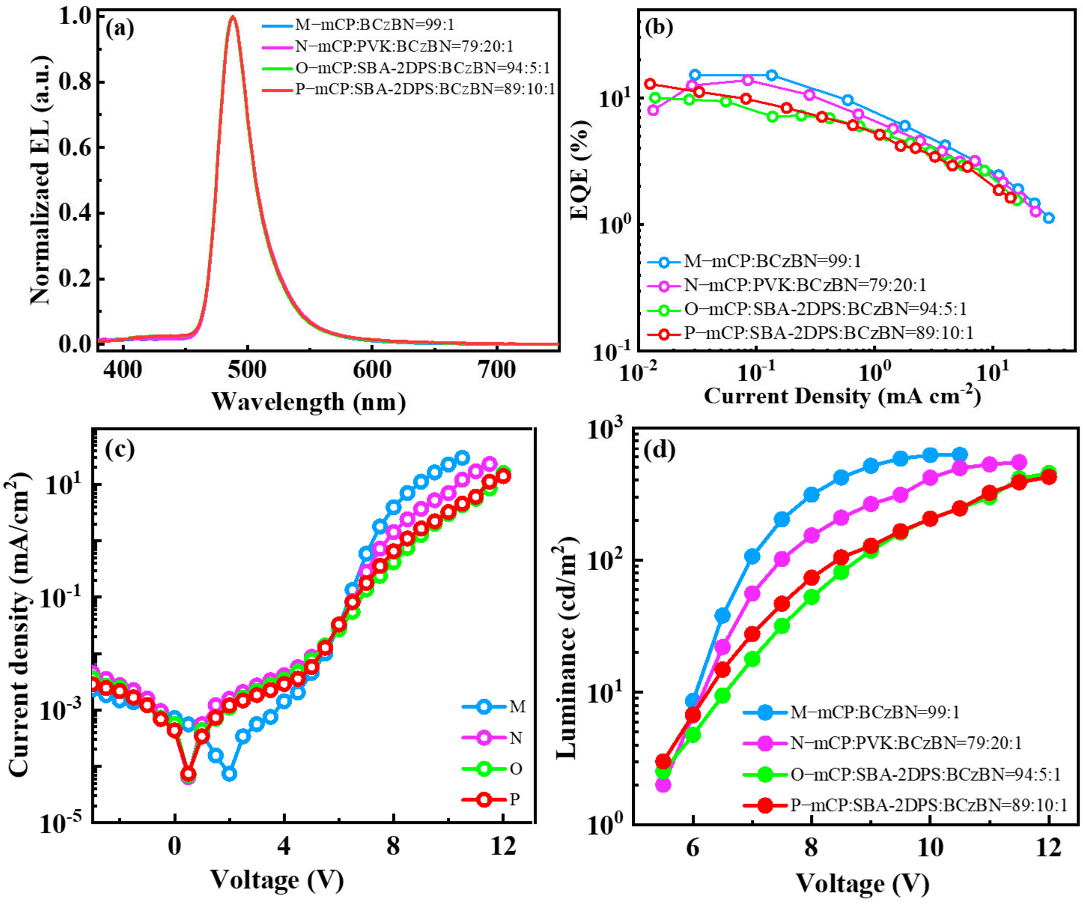

Figure S9. (a) Normalized EL spectra of the spin-coated devices. (b) External quantum efficiency versus current density curves for the spin-coated devices. (c) Current density−voltage curves. (d) Luminance−voltage curves.

Table S3. Comparison of the EL Performances of the spin-coated devices with different EML: mCP:BCzBN = 99:1 for Devcie M, mCP:PVK:BCzBN = 79:20:1 for Devcie N, mCP:SBA-2DPS:BCzBN = 94:5:1 for Devcie O, and mCP:SBA-2DPS:BCzBN = 89:10:1 for Devcie P.

device	λ_{max}[nm]	FWHM[nm]	V_{on} ^{a}[V]	CE_{max}^{b} [cd A^{-1}]	EQE_{max}^{c} [%]	CIE(x,y)^{d}
M	488	31	6.2	28.6	15.3	(0.11,0.38)
N	488	31	6.3	26.0	13.8	(0.11,0.38)
O	488	31	6.6	18.3	10.1	(0.11,0.36)
P	488	31	6.4	23.9	13.0	(0.11,0.37)

[a]Driving voltage at the brightness of 10 cd/m^2. [b]Maximum of current efficiency. [c]Maximum of external quantum efficiency. [d]The Commission Internationale de I'Eclairage 1931 color coordinates.

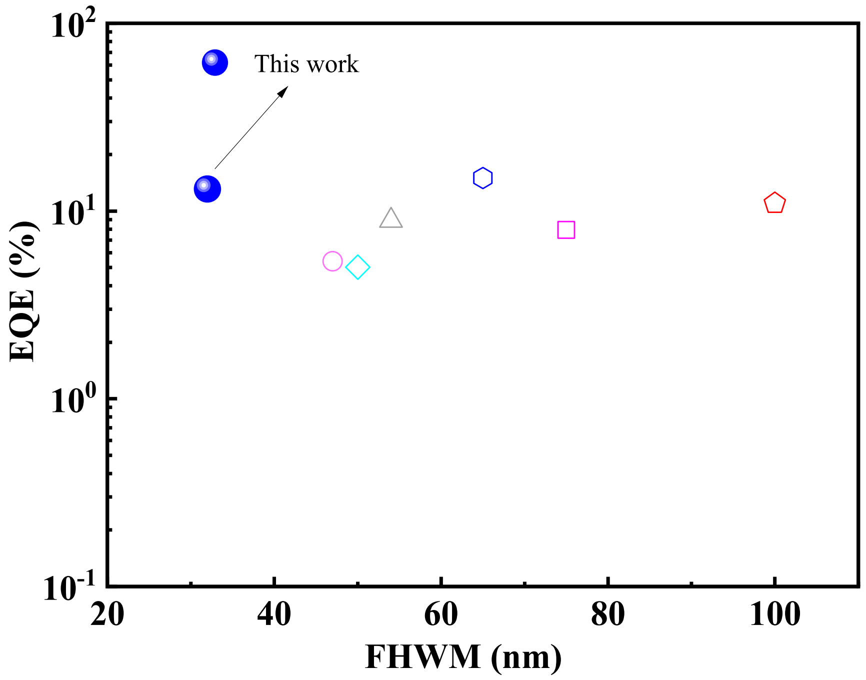

Figure S10. Figure of merit of EQE and FWHM of OLEDs based on inkjet printing reported in literature.

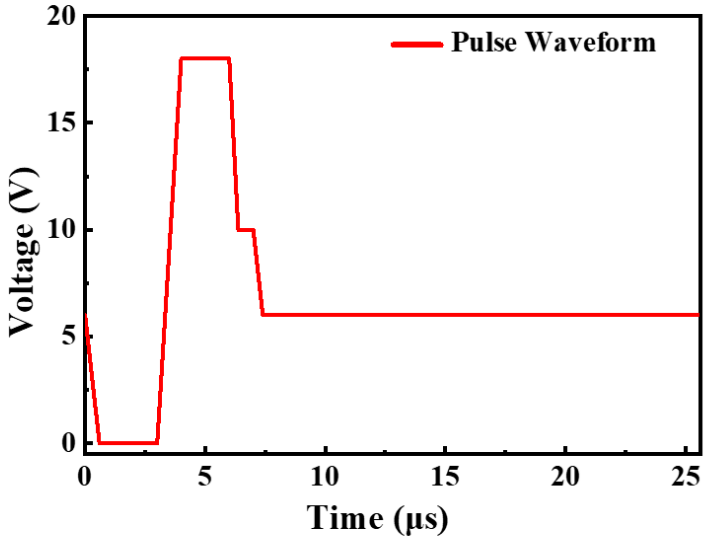

Figure S11. Inkjet printing waveform.
